# Mechanisms of allergen-specific immunotherapy for allergic rhinitis and food allergies

**DOI:** 10.1042/BSR20200256

**Published:** 2020-03-31

**Authors:** Hiu Yan Lam, Vinay Tergaonkar, Kwang Seok Ahn

**Affiliations:** 1 Laboratory of NF-κB Signaling, Institute of Molecular and Cell Biology (IMCB), 61 Biopolis Drive, Proteos, Singapore 138673, Singapore; 2 Department of Biochemistry, Yong Loo Lin School of Medicine, National University of Singapore (NUS), Singapore 117596, Singapore; 3 Department of Pathology, Yong Loo Lin School of Medicine, National University of Singapore (NUS), Singapore 117596, Singapore; 4 Department of Science in Korean Medicine, Kyung Hee University, 24 Kyungheedae-ro, Dongdaemun-gu, Seoul 02447, Republic of Korea

**Keywords:** allergen immunotherapy, allergic rhinitis, allergies, food allergies

## Abstract

Allergen-specific immunotherapy (AIT) is currently the only potential treatment for allergies including allergic rhinitis (AR) and food allergies (FA) that can modify the underlying course of the diseases. Although AIT has been performed for over a century, the precise and detailed mechanism for AIT is still unclear. Previous clinical trials have reported that successful AIT induces the reinstatement of tolerance against the specific allergen. In this review, we aim to provide an updated summary of the knowledge on the underlying mechanisms of IgE-mediated AR and FA as well as the immunological changes observed after AIT and discuss on how better understanding of these can lead to possible identification of biomarkers and novel strategies for AIT.

## Introduction

The prevalence of allergy is increasing, and the most common manifestation of allergy is respiratory allergic diseases such as allergic rhinitis (AR) [1]. AR is an inflammatory disorder of the nasal mucosa to the allergen mediated by Immunoglobulin E (IgE) and affects approximately 25% of the population worldwide and hence is the most common form of allergy [2]. Allergen causes the inflammation of nasal cavity membrane in AR and the common symptoms for AR include sneezing, itching, rhinorrhea, nasal congestion, erythema and tearing eyes, causing sleep impairment as well as reduced performance at work and school and therefore significantly affect the quality of life for patients with AR and the cost to society is substantial due to the high prevalence [1]. There are two types of AR, seasonal and perennial. Seasonal AR occurs due to the presence of seasonal allergens such as grass pollens and tree pollens while perennial AR occurs throughout the year and is triggered by non-seasonal allergens, including animal dander and house dust mite (HDM) [3].

Currently, clinical guidelines recommend a combination of allergen avoidance, pharmacotherapy and/or allergen-specific immunotherapy (AIT) for the treatment of AR [1]. Pharmacotherapy, although relatively safe and inexpensive, can only target the allergic symptoms without having any effect on the disease progression and therefore, pharmacotherapy is required to be repeatedly administrated so long as symptoms prevail, meaning that it is usually life-long [1]. Moreover, many patients report that these pharmacotherapies are insufficient to control their allergic symptoms [1]. On the other hand, AIT is the only disease-modifying and curative therapy for AR, with the aim to induce persistent immunological tolerance against the allergen of interest. AIT usually involves administering gradually increasing dose of the specific allergens to the allergic patients until the effective dose is reached which takes about several weeks and this is then followed by administering the maintenance doses for at least three years [4,5] and each dose of allergen administered in AIT is approximately 100 times the maximum year intake through natural exposure [1]. When given continuously for three years, recent guidelines have indicated the effectiveness of AIT in patients with IgE-dependent AR, in particular those with seasonal AR, and persistent clinical benefits are observed in these patients for several years after discontinuation of the immunotherapy [6].

In addition, a pandemic increase in non-respiratory allergies such as food allergies (FA) has also been observed [7–11] and patients with FA have a higher risk of developing respiratory allergies [12–14]. FA is defined as an adverse immunological reaction to food [8,15], which may be IgE-mediated, non-IgE mediated (cell-mediated) or mixed (IgE and cell-mediated). IgE-mediated food reactions usually occur within minutes to 2 hours after exposure to allergen [16,17] while non-IgE-mediated food reactions are typically delayed from hours to weeks after intake of the allergen [18]. FA are diagnosed based on detailed clinical history and physical examination together with skin-prick test or serum-specific IgE testing, elimination diet for diagnostic purposes and oral food challenge [19]. For non-IgE-mediated FA, patients will have negative results for skin-prick test or specific IgE but positive for oral food challenge or responsive to a trial of elimination of the suspected offending food [18]. In this review, we will focus on IgE-mediated FA, which has been studied and documented much more in-depth than non-IgE-mediated FA. The self-reported prevalence of FA is about 15% in adults but the prevalence of FA in adults is 1% as reported in studies that used more stringently defined criteria [20]. In the EuroPrevall birth cohort study which 823 infants in the UK were followed up until 2 years old, the cumulative incidence of FA was 5% and about half of these infants were with non-IgE-mediated FA [21]. Indeed, as published in the “Finding a path to safety in food allergy: assessment of the global burden, causes, prevention, management and public policy” by the National Academies of Sciences, Engineering, and Medicine (NASEM), it is difficult to assess the true prevalence of FA [22]. The quality of life of children with FA and their family is significantly reduced due to dietary and social restrictions, severe reactions, and fatalities that might be caused by FA [8,23]. Current management of an IgE-mediated FA is limited to allergen avoidance, patient education on prompt recognition of symptoms and emergency treatment of allergic reactions [8]. However, there is low compliance to diet adherence [24]. Based on a similar concept to AIT for AR, AIT has been introduced to FA patients for managing their FA recently with an aim to restore immune tolerance to the food allergen and has been considered the most promising therapeutic approach for treating FA [8]. Indeed, the Food and Drug Administration has only recently approved OIT for peanut allergy with the use of AR101, a characterized and standardized peanut powder, in children and adolescents with peanut allergy after a previous phase 3 clinical trial (PALISADE) has demonstrated that treating children and adolescents with severe peanut allergy with AR101 resulted in desensitization after 6 months of the maintenance phase in these allergic individuals [25]. However, unlike AIT for AR, there is currently lack of evidence that AR101 can induce long-term tolerance after discontinuation with the OIT [25].

Despite AIT has been applied for more than 100 years since the first scientific publication by Leonard Noon in 1911 in *The Lancet*, the immunological mechanism for AIT is still not fully understood. Moreover, even having met the selection criteria for receiving AIT, a significant proportion of patients do not respond to the treatment [5] and there is currently no clear biomarker which can invariably predict clinical response to AIT [26,27]. Hence, in this review, we will outline the immunological mechanisms of AIT for AR and FA. Through better understanding of the underlying mechanisms in the induction of tolerance, it will enable us to develop novel strategies for AIT with improved efficacy and safety, as well as to identify potential biomarkers which allow prediction of individual success before or during early stage of the treatment.

## Immunological mechanisms of allergic rhinitis

Within the upper respiratory tract of human, there is a dense network of antigen presenting cells, comprising monocytes, macrophages and dendritic cells (DCs). DCs, being the most effective cells for the induction and regulation of primary immune response, sit in the paracellular and intercellular channels around the basal epithelial cells [28].

Sensitization occurs when an allergen is inhaled and passes through the inflamed nasal epithelium. As a result, C-C motif chemokine ligand 2 (CCL2) and CCL20 are released by activated epithelial cells to recruit immature DCs. The cytokines, interleukin-25 (IL-25) and IL-33 as well as thymic stromal lymphopoietin protein (TSLP) expressed in the respiratory epithelium of atopic allergic subjects favor the development of pro-allergic DCs, which provides help for T helper 2 (Th2) cell differentiation [6,29,30]. Group 2 innate lymphoid cells (ILC2s) that amplify and maintain local Th2-driven allergic inflammation also rely on these epithelial-derived cytokines as their growth factors [6,26,31,32]. IL-13 produced by ILC2s and IL-4 produced by basophils lead to the migration of DCs to regional draining lymph nodes where they polarize naïve T helper cells into T helper 2 (Th2) cells [6]. Cytokines including IL-4 and IL-13, which promotes the production of antigen-specific IgE by B cells, are further produced by Th2 cells. Apart from Th2 cells, follicular helper T (Tfh) cells originated from the germinal centre of the lymph node are another source of IL-4 that contributes to the class-switching to IgE in B cells [6]. During isotype switching, certain B cell subsets become plasma cells, switching from IgM to IgE production and these IgE binds to the high-affinity receptor for IgE (FcεRI) expressed on the surface of mast cells (MCs) and basophils, resulting in sensitization [33,34].

When the individual is exposed to the same antigen again, IgE–FcεRI complexes are cross-linked by allergens and this IgE cross-linking on MCs and basophils causes the preformed mediators within the secretory granules of MCs and basophils such as histamine and tryptase to be released within seconds or minutes, and such process is known as degranulation and characterized the early-phase response or type I immediate hypersensitivity reaction [35]. Newly synthesized lipid mediators such as leukotrienes (LTC4, LTD4, LTE4) and prostaglandins (e.g. PGD2) will also be produced by MCs [3,6] and these lipid mediators help to sustain the inflammation through attracting neutrophils and eosinophils to the site of insult [3]. As a result, vasodilation, edema, mucus secretion, bronchial smooth muscle contraction and acute bronchoconstriction are observed during the immediate type I hypersensitivity [6].

Hours after challenge, MCs also *de novo* synthesize and release pro-inflammatory cytokines (eg. IL-6, tumor necrosis factor α (TNFα)) and Th2-type cytokines (eg. IL-4, IL-13) and chemokines (eg. CCL3) orchestrated by several transcription factors [33,36–44] including nuclear factor-κ B (NF-κB) which is the master regulator of immunity, inflammatory response [45–47] and cell survival [48–53]. For instance, expression of IL-6 in activated MCs is mediated by NF-κB [54] while that of TNFα and IL-13 in activated MCs requires nuclear factor of activated T cells (NFAT) [55]. In addition, c-Fos of the activator protein-1 (AP-1) complex is involved in the transcription of several cytokines in MCs including IL-6, TNFα, IL-13 and chemokine CCL3 upon IgE-mediated activation [56]. Eosinophils are also recruited to the respiratory mucosa in response to the IL-5 produced by Th2 cells and ILC2 [5] and tissue eosinophilia and nasal congestion will occur [6]. Eosinophils are essential in the allergic cascade and they produce oxygen free radicals such as hydrogen peroxide and superoxide anion, damaging the epithelium as well as promoting intense inflammatory response [3] and leading to the activation of diverse signaling cascades [57,58]. Further, neurotoxin derived from eosinophil together with eotaxin induced by eosinophil and the granular products of eosinophils such as eosinophil cationic protein are important in generating nasal hyper-reactivity and severe damage can be caused to the epithelium of the respiratory tract and exposing local nerve fibers [3]. This process can interrupt the production of natural endopeptidase, the neuropeptides released from the nerve fibers are thereby not degraded and this prolongs the inflammatory response [3]. Such late phase response can sustain for days or even weeks for some patients after a single nasal allergen challenge [6].

## Immune basis of food allergy

Sensitization to food can happen through the oral route but sensitization can also occur through the skin, or occasionally through the airways. In the context of FA, the development of regulatory T (Treg) cells is compromised and instead there is polarization of the Treg cells toward the Th2 phenotype characterized by IL-4 production [59]. Peripheral blood mononuclear cells (PBMCs) from milk-allergic children after culturing with milk β-lactoglobulin for 5 days and restimulation with PMA and ionomycin have elevated IL-4 and IL-13 expression in their milk-specific Tregs comparing with PBMCs from control subjects or from peanut-allergic children without milk allergy [59,60]. Besides Th2 cells, ILCs, basophils [61,62] and T follicular helper cells (Tfh) [59,63] also secrete IL-4 to further enhance the type 2 immune responses. The IL-4 produced drives B-cell class switching to IgE and these allergen-specific IgE (sIgE) is crucial to the development of food allergies [64].

Eczema has long been identified as a major risk factor for FA [64–66] and indeed, growing evidence have indicated that skin is the main site of sensitization to foods, particularly peanut, in infants. For instance, the study by Lack et al*.* [67] demonstrated an association of peanut allergy in infants after the use of diaper creams which contains peanut oil while another study by Fukutomi et al*.* [68] showed that the use of facial soap containing wheat protein leads to a higher risk for wheat allergy [64]. Filaggrin is a protein important for maintaining the skin barrier and mutation in this protein is associated with increased risk of atopic dermatitis as well as FA [64,69,70]. Moreover, Brough et al*.* [71] demonstrated that children with filaggrin mutation have higher risk of peanut sensitization that is associated with early life environmental peanut exposure, supporting the hypothesis that peanut allergy can develop in children having an impaired skin barrier through skin sensitization. Furthermore, T cells from peanut-allergic patients that express cutaneous lymphocyte antigen (CLA), a skin-homing marker, have enhanced proliferative capacity to peanut comparing with those α4β7 integrin (gut-homing) expressing T cells [64,72]. Additionally, Abernathy-Carver et al*.* [73] reported similar findings in young children with atopic dermatitis and milk allergy. Hence, skin might be the primary site for sensitization to peanut and milk.

IL-33 that is elicited through the skin has also been demonstrated to be important for FA as topical application of peanut leads to innate stimulatory activity on human DCs [64,74]. Sensitization can also be promoted by TSLP through DCs and basophils [64,75,76]. DCs that express OX40L induce IL-3-secreting CD4^+^ T cells and recruit basophils into the draining lymph nodes and the basophils produce IL-4 as well as promote IL-4 production by cluster of differentiation 4 (CD4)^+^ T cells [64,77]. Moreover, IL-9 has also been implicated as a key cytokine for allergic response to foods and IL-9 was the highest differentially expressed gene in peanut-activated memory CD4^+^ T cells from peanut-allergic children comparing with those from children without peanut allergy [64,71] while IL-9 was also secreted by PBMCs from egg-allergic children but not from non-allergic children after stimulation with egg white protein [64,78]. Further, IL-9 is a growth factor for MCs and a population of mucosal MCs that produces a high amount of IL-9 and IL-13 is associated with the symptoms observed after oral challenge while elevated gene expression levels of IL-9 and IL-13 were detected in the duodenum of FA subjects comparing with healthy controls [64,79].

Natural oral tolerance involves active suppression of antigen-specific immune responses induced in the gastrointestinal tract and such oral tolerance requires robust induction of Treg cells within the mucosa and is initiated by CD103^+^ DCs, which capture antigen in the lamina propria and migrate to the mesenteric lymph nodes. In response to antigen presentation by the CD103^+^ DCs under a transforming growth factor β (TGF-β) and retinoic acid-dependent mechanism, naïve T cells differentiate to Tregs [59,80–82]. Retinoic acid induces expression of C-C motif chemokine receptor 9 (CCR9) and α4β7 gut-homing markers by T cells [59,83] and gut-homing markers expressing antigen-specific Tregs migrate from the lymph nodes to the lamina propria, where they expand following production of IL-10 by C-X3-C motif chemokine receptor 1 (CX3CR1)^+^ macrophages [59,84]. Goblet cell-associated antigen passages (GAPs) in the intestinal epithelium forms to deliver antigen exclusively to the tolerogenic CD103^+^ CXC3CR1^−^DCs, favoring the induction of Tregs. Since the frequency of GAPs is increased by the mucin production from goblet cells, antigen delivery is enhanced [59,85]. Moreover, hyperglycosylated mucin MUC2 imprints CD103^+^ DCs with a regulatory phenotype through inducing the expression of TGF-β, retinaldehyde dehydrogenase (RALDH) and IL-10 and suppressing inflammatory cytokines, thereby promoting oral tolerance. Indeed, increase in Treg cells and IL-10-producing CD4^+^ T cells after antigen stimulation were observed in children who have naturally outgrown their FA as compared with children who have active allergy or non-allergic controls [64,86], suggesting an important role of Tregs in the development of food tolerance.

Several epidemiology studies have demonstrated that consuming food such as peanut, milk, fish or wheat in early life has been associated with reduced incidence of FA [64,87–90] and randomized controlled trials have been conducted to evaluate the development of FA as a result of early introduction of foods. Lerodiakonou et al. [91] showed in their meta-analysis of randomized controlled trials that early introduction of egg or peanut to the diet was associated with a lower risk of developing egg or peanut allergy respectively. The LEAP trial showed that early introduction of peanut to the diet of children with increased risk of peanut allergy between the ages of 4 months and 11 months resulted in a dramatic reduction in the prevalence of peanut allergy as compared with those children who did not receive peanut and such protection was maintained even after the cessation of peanut consumption for 12 months and induction of peanut-specific IgG4 was observed as a result of peanut inclusion in the diet [92]. For egg allergies, the PETIT trial yielded positive results in a high-risk cohort of 4- to 6-month-old children with eczema after introduction of egg to their diet and showed a significant increase in ovomucoid-specific IgG4, IgG1 and IgA and diminished ovomucoid-specific IgE following introduction of egg into their diet at a very young age [93]. Yet, the studies by Bellach et al*.* [94] and Palmer et al*.* [95] demonstrated that a significant proportion of children who were already sensitized and clinically active to egg at 4-month-old poorly tolerated the early introduction of egg into their diet and therefore more randomized controlled trials are needed to support the tolerogenic nature of oral exposure to egg at early age.

## Mechanisms of allergen-specific immunotherapy

AIT helps the immunity to restore its normal function against specific allergens in long term by inducing specific allergen tolerance [96] and is currently recognized as the only clinically effective treatment with disease-modifying effect for AR [97]. Clear evidences from various studies have shown that 3 years of AIT treatment for seasonal AR patients results in long-term remission of symptoms for several years after treatment discontinuation [98–101]. Although only sustained unresponsiveness but not permanent tolerance has been demonstrated in AIT for FA [27], AIT is still considered to be the most promising treatment for patients with FA [59,64].

AR patients who experience difficulties sleeping due to symptoms or those who have symptoms that affect their performance at school or work despite the use of pharmacotherapy and allergen avoidance measures might be considered for AIT treatment [102]. AIT for AR can be administered subcutaneously (SCIT). SCIT is injected to patients at an increasing dose each week for several weeks, followed by monthly maintenance injections for at least 3 years and SCIT is performed at a specialist allergy clinic that is equipped to manage life-threatening anaphylaxis. Sublingual immunotherapy (SLIT) has emerged as an alternative route of desensitizing AR patients and involves daily drops or tablets of allergen extract placed under the tongue and can be self-administered by the patient at home and offers a more favorable safety profile over SCIT. Both SCIT and SLIT have been shown to be effective in AR [98,102], especially seasonal AR. Indeed, in a trial by Bozek et al*.* [103] where patients were prospectively observed for 20 years after completion of AIT against pollen, prolonged clinical effect was confirmed in these patients.

The most common and earliest FA in infants are milk and egg allergies [64]. Most milk- or egg-allergic children can tolerate heat-denatured milk and egg proteins and incorporating baked-milk and/or baked-egg products into the diets of these children has facilitated the development of full tolerance to all forms of milk and egg in them [104,105]. Since then, including heat-denatured milk and/or egg products into the diet of young children who can tolerate these products early has become a standard practice by clinicians [106]. Various routes of administration of AIT for FA including oral (OIT), epicutaneous (EPIT), sublingual (SLIT) and subcutaneous (SCIT) routes have been investigated, with OIT being most studied [64] while SCIT for FA using unmodified food extracts was abolished due to severe anaphylactic side effects despite efficacy being shown [107,108]. During AIT for FA, the allergic individual is exposed to gradually increasing doses of the food allergen over several years, with an aim to achieve sustained unresponsiveness to the food allergen even after stopping treatment (i.e. remission) [64]. The term ‘remission’ instead of ‘tolerance’ is used for FA as clinical reactivity usually recurs in a time-dependent manner in FA patients after AIT [64], although there are increasing evidences showing remission in a subset of FA patients after discontinuation of AIT [78,106,109–114]. FA patients who undergo OIT are given a very low dose of allergen at the beginning of the treatment and the dose of allergen is gradually increased over a period of months to reach a maintenance dose (300 mg to low gram daily) and similar to AIT for AR, the whole AIT for FA usually requires years to achieve remission [64]. SLIT and EPIT are administered to FA patients as drops under the tongue or via skin patch respectively and the allergen doses for SLIT and EPIT (1–10 mg for SLIT and 100–250 μg for EPIT) are lower than that of OIT and the lower dose used in SLIT and EPIT has contributed to a better safety profile but reduced efficacy than OIT [64]. AIT inhibits the early- and late-phase allergic responses during AR and FA by inducing immunological tolerance through diverse mechanisms.

## The impact of allergen-specific immunotherapy on T cells

T-regulatory (Treg) cells plays a key role in the immune tolerance to allergen after AIT [115–119]. There are two main types of Tregs, natural regulatory T (nTreg) cells that express forkhead box P3 (FOXP3)^+^ CD4^+^ CD25^+^ and developed in the thymus and present in birth, as well as the inducible Treg (iTreg) cells generated in the periphery under different tolerogenic conditions which produce regulatory cytokines such as IL-10 and transforming growth factor β (TGF-β). Different studies have shown elevated number of nTregs after AIT for AR. For instance, study by Radulovic et al*.* [120] showed an increase in nTregs in the nasal mucosa of SCIT-treated AR patients comparing with untreated controls while the study by Rosser et al*.* [121] demonstrated an increase in nTregs in the sublingual biopsy specimens from AR patients who have undertook SLIT. Although induction of Tregs is observed in natural oral tolerance, there are discrepancies in the role of Tregs in the development of tolerance after AIT for FA. One study by Varshney et al. [122] demonstrated that peanut OIT is associated with increased ratio of FOXP3 high: FOXP3 intermediate CD4^+^ CD25^+^ T cells at the time of oral food challenge while the study by Bedoret et al*.* [123] showed that sustained tolerance seen in milk-allergic patients who have received OIT with omalizumab was due to anergy of T-cell response, with no evidence of antigen-specific Tregs being produced in these patients. Interestingly, epigenetic changes are induced by AIT. Epigenetic modification at the FOXP3 promoter region that affects the Treg function was investigated in a randomized controlled study for SLIT. The participants within the study were having allergy to both grass pollen and HDM, and reduced methylated CpG sites within the FOXP3 locus of enriched peripheral memory Treg cells was reported after 12 months of treatment, leading to the suppressive function and long-lasting production of Tregs [124–126]. Likewise, hypomethylation was also observed in the FOXP3 CpG sites in antigen-induced Tregs in OIT-treated patients with peanut allergy [126].

In AIT, high levels of anti-inflammatory cytokines IL-10 and TGF-β are produced by the autocrine action of allergen-specific Treg cells and this initiate peripheral T-cell tolerance [115–119,127]. The increased number of IL-10-expressing T cells during pollen season and the seasonal increase in TGFβ^+^ T cells was correlated to the increase in serum IgG4 levels [128] and the increase in peripheral circulating IgA concentration, respectively [129]. In the study by Francis et al. [130], PBMC collected from patients with grass pollen allergy at week 2–4 during the early up-dosing phase for SCIT and co-cultured with grass pollen allergen for 6 days released high levels of IL-10 and demonstrated repressed late-phase response. Similarly, the study on HDM AR patients conducted by Boonpiyathad et al*.* [131] showed elevated number of Der p1-specfic FOXP3^+^ Helios^+^ IL10^+^ Tregs but diminished ILT3^+^ Treg cells were correlated to improved allergic symptoms in these patients after SCIT. Further, the study by Syed et al*.* [126] demonstrated the association between development of sustained tolerance after OIT and the induction of IL-10-expressing antigen-specific Tregs after peanut stimulation [59].

Besides IL-10 and TGF-β, iTregs can also produce IL-35 [6], a newly identified inhibitory cytokine belonging to the IL-12 family of heterodimeric cytokines that elicit anti-inflammatory response [132]. Shamji et al. showed that IL-35 produced by iTregs suppresses type 2 immune response mediated by ILC2s and Th2 cells in AR patients allergic to grass pollen and their further results discovered that allergen-driven IL-35 levels and cell counts for IL-35-producing Tregs were higher in AR patients receiving SLIT and non-atopic controls comparing with patients with seasonal AR without SLIT treatment, indicating IL-35 and IL35-producing Tregs as important immune regulators induced by SLIT [132]. Together, these findings demonstrate that regulatory cytokines secreted by allergen-specific Tregs during AIT dampen the allergic response.

Recently, a novel effector subset of regulatory T (Treg) cells, follicular regulatory T (Tfr) cells have been identified. Tfr cells can suppress Tfh cell-mediated B-cell activation and antibody production and share some phenotypic characteristics with both Tfh and Treg cells [133,134]. The study by Yao et al*.* [135] showed that there were increased numbers of circulating Tfr cells with improved suppressive function in AR patients after HDM-specific SCIT. Therefore, better understanding of Tfr cells will benefit the development of novel strategies for AIT.

## Reduced Th2 cells and shifting of Th2 to Th1

Apart from the effect seen in Treg cells, AIT has also been shown to result in the reduced number of Th2 cells in nasal mucosa [136] and decreased Th2 cytokines in the nasal lining fluid was also observed after nasal challenge [137]. In the GRASS trial by Scadding et al. [98], both SCIT and SLIT led to a decreased number of peripheral chemoattractant receptor—homologous molecule expressed on Th2 lymphocytes (CRTH2)^+^ CCR4^+^ CD27^−^ CD4^+^ Th2 cells and decrease in Th2 cytokines including IL-4, IL-5 and IL-13 in the nasal fluid after nasal allergen provocation at 2 years of continuous AIT and these changes were associated with improved clinical symptoms. Similarly, the number of IL-5^+^ IL-13^+^ CD27^−^ CD161^+^ CD4^+^ cells and ST2^+^ CD45RO^+^ CD4^+^ Th2 cells dropped significantly in HDM-allergic patients after 52 weeks of SLIT [138]. Moreover, shift of the immunity from Th2 toward Th1 immune response is also demonstrated after AIT. The mRNA for interferon γ (IFNγ) in T cells from nasal mucosa is up-regulated after allergen challenge and is related to the reduced nasal symptoms during pollen season [100]. There is also increased IFNγ protein levels in the nasal fluid of pollen immunotherapy-treated patients during natural seasonal allergen exposure [139]. In addition, macrophages from the skin express increased IL-12 mRNA after grass pollen SCIT and is positively correlated to local IFNγ^+^ T cells and inversely correlated to IL-4^+^ T cells [140]. Hence, decreased Th2 cell numbers and polarization of Th2 to Th1 immune response have been linked to the development of peripheral tolerance in AR patients who have undergone AIT.

## Influence on antibody synthesis and regulatory B cells

Both SCIT and SLIT for AR have been shown to cause an early but transient increase in serum allergen-specific IgE (sIgE) and reduced back to the pre-treatment levels during the maintenance phase [6,98,141]. AIT induces increase in serum allergen-specific IgG (sIgG), in particular IgG4 (sIgG4) and IgG1 (sIgG1), in 10- to 100-fold [142–144] and has been correlated to clinical outcomes [143]. Similar to the results from AIT trials for AR, there is an initial rise in sIgE followed by a steady decline, as well as increased sIgG4 are observed after AIT for FA [59,109,145,146]. It is worth noting that the level of IgG4 drop back to pre-treatment level within 1 year after discontinuation of AIT, although the inhibitory capacity to serum IgE by IgG4 persists several years together with clinical benefits [6,147], suggesting that these IgG4 antibodies might have either higher avidity or higher affinity [6,64]. Such hypothesis is supported by the enhanced somatic mutation in sIgG4 but not IgE during OIT [64] and the long-term tolerance is probably due to long-lived memory B cells induced by the AIT [6].

The serum sIgG produced after AIT can compete with sIgE for allergen binding, thereby blocking allergen–IgE complex formation and preventing MC and basophil degranulation, IgE-dependent cytokine secretion from MCs [96], binding of allergen to the B-cell receptor on IgE^+^ memory B cells [148], as well as allergen presentation to T cells [149]. Importantly, a recent paper by Shamji et al*.* [150] demonstrated that nasal IgG4 level was up-regulated in the SCIT patients comparing to that without SCIT during the pollen season compared with out of season and IgG- associated inhibitory activity in nasal fluid and serum was significantly enhanced in the SCIT group comparing with the seasonal AR patients without SCIT treatment and such blocking activities were correlated to global symptom improvement, demonstrating that sIgG4 produced locally at the nasal mucosa can be a potential biomarker for immunotherapy efficacy. Similarly, the study by Kim et al*.* [151] showed an increase in serum sIgG4 levels together with improved total nasal symptom score for HDM allergic AR patients after undertaking SLIT. The blocking effect of sIgG4 has been confirmed by a recent clinical trial which showed that single application of two blocking human monoclonal cat allergen Fel d 1-specific IgG antibodies led to reduced symptoms upon nasal challenge and the magnitude of such reduced symptoms was comparable to that observed after years of AIT [148,152]. Apart from IgG, allergen-specific IgA (sIgA) is also induced during grass-pollen SLIT [6,101,130,153,154], HDM SLIT [151] and egg OIT [64,155]. Previous study has associated deficiency in IgA with increased risk of FA [61,156] but more work is needed to understand the role of IgA in inhibiting allergic reactions after AIT.

In addition, an *in vitro* study has shown that crosslinking of FcγRIIb by IgG4 redirects M2a pro-allergic macrophages to an M2b-like immunosuppressive phenotype, as seen by the down-regulation of CD163 and CD206 but significantly up-regulated secretion of IL-10, IL-6, TNFα and CCL1 [157], suggesting the possibility of driving macrophages from the pro-allergic toward the immunosuppressive phenotype as an alternative approach for treating AR patients.

The involvement of B cells in allergen tolerance is not as well explored as that of T cells but the few studies have implicated the role of B cells in AIT, mainly through the regulatory B cells (Breg), a subset of B cells that have immunosuppressive and anti-inflammatory properties, predominantly via the release of IL-10, supporting Treg differentiation, the increase in IgG4 production and inhibition of the pro-inflammatory responses mediated by T cells and DCs. IL-10-producing Bregs isolated from bee venom-tolerant subjects has high expression of CD25 and CD71 but low expression of CD73 on their cell surface and these cells are capable of suppressing the proliferation of bee venom-specific T cells [158]. Three to four months after the start of venom immunotherapy, there is 2- to 5-fold increase in the number of phospholipase A2 (PLA)-specific IL-10-producing Bregs, and the level is comparable to that of healthy bee keepers during the season [159–161]. Interestingly, both groups have high levels of PLA-specific IgG4-switched memory B cells, plasmablasts and PLA-specific CCR5-expressing B cells [162]. Additionally, allergen-specific IgG4 were also produced by IL-10^+^ Breg cells after immunotherapy for bee venom. Similarly, circulating allergen-specific B cells has also been observed after peanut immunotherapy [163]. Recently, the study by Shamji et al*.* [150] showed for the first time that grass pollen SCIT led to an increase in the number of IL-10^+^ Breg cells that associates with the increase in sIgG4 levels in the nasal fluid and the inhibitory activity caused by these sIgG4 correlates closely with the clinical response for SCIT. Thus, these findings suggest a potential role of Breg cells in inducing the tolerance against aeroallergen and more work should focus on the characterization of Breg cells after AIT for AR in the future.

## Effects on MCs, eosinophils, basophils, ILC2s and DCs

Both SCIT and SLIT suppress early- and late-response after allergen challenge [164,165] and less histamine and tryptase are released in the nasal fluid during the early phase response while reduced number of eosinophils [166] and diminished Th2 cytokine levels such as IL-4, IL-5 and IL-13 [137] during the late phase response were observed, probably due to reduced activation of MCs and basophils as a result of the blocking activity of sIgG4 produced that compete with sIgE for allergen after AIT [167]. Further, a double-blinded clinical trial for grass pollen SCIT has shown reduced number of MCs, basophils and eosinophils in the nasal mucosa of SCIT-treated participants as compared with pre-treatment [168,169]. In the study by Shamji et al. [170], reduced basophil responsiveness and histamine release, through the quantification of intracellular histamine-binding fluorochrome-labeled diamine oxidase and measurement of CD63, CD203c and CD107a cell surface expression, were reported in whole-blood basophil from AR patients after both SCIT and SLIT and the result was correlated to lower symptom scores. Increased tolerability of basophils to Parietaria allergen (CD-Sens measured by CD63 and CD203c) and a significantly reduced symptom severity were observed in AR patients treated with SLIT [171]. Further, significantly decreased basophil activation was also observed after peanut OIT (Peanut oral immunotherapy study: safety, efficacy and discovery - POISED study) and this is correlated to higher sIgG4/ sIgE ratio and the study suggested that patients with low basophil responsiveness were more likely to achieve treatment success to peanut OIT [172]. Similarly, there was significant decrease in basophil sensitivity to Ara h 2 peanut allergen in patients with sustained unresponsiveness after peanut OIT [173]. Hence, these studies suggest that basophil responsiveness can be a useful biomarker to identify allergic patients more likely to achieve treatment success after AIT.

Adhesion and chemotactic factors for eosinophils reduces after AIT and this is related to the diminished bronchial hyper-reactivity [174]. Eosinophil activity and function are down-regulated by the IL-10 produced from the increased numbers of iTregs during AIT. Besides, the IL-10 also suppresses the production of IL-5 from Th0 and Th2 cells [175], which has a direct correlation to the number of nasal mucosal eosinophils and decrease in eosinophil number lessens the severity of symptoms for AR. Furthermore, those IL-10 produced by iTregs can also lower the density of MCs, reduce the concentration of local histamine in addition to preventing the growth and degranulation of MCs [176]. Thus, AIT reduced the recruitment and/or activation of effectors cells at allergic tissue sites.

Growing evidences have demonstrated the role of ILC2s in allergic diseases and therefore there is increased focus on how AIT modulates this group of immune cells to induce tolerance in allergic patients. Lao-Araya et al. [177] showed that SCIT-treated patients had suppressed peripheral ILC2s that correlated with the severity of self-reported symptoms during the pollen season while Fan et al*.* [178] reported a reduction of ILC2s in the peripheral blood of SCIT-treated, HDM allergic AR patients compared with that of the untreated group. Furthermore, the study by Mitthamsiri et al*.* [179] showed that HDM AR patients responding to SCIT and healthy individuals had less circulating ILC2s comparing with non-responders and AR patients. These studies suggest ILC2s as a potential biomarker for the therapeutic effect of AIT for AR.

**Figure 1:**
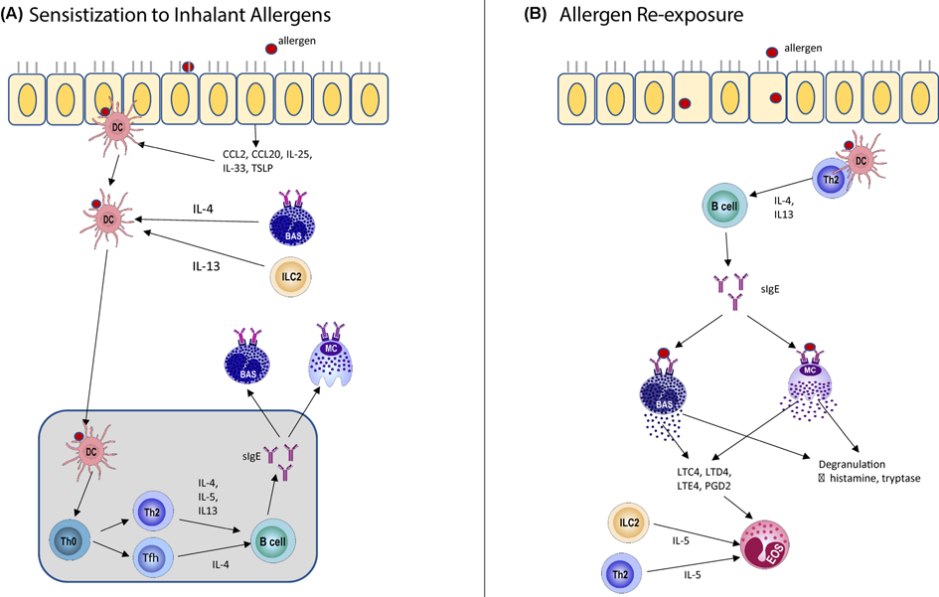
Immune mechanisms of (A) initial sensitization after inhaling the allergen and the (B) immune responses after subsequent encounters with the same inhalant allergen (**A**) Nasal epithelium releases CCL2 and CCL20 which recruit immature DCs. Epithelial cytokines including IL-25, IL-33 and TSLP favor the development of pro-allergic DCs. Activated DCs migrate to the lymph nodes after IL-4 production by basophils and IL-13 production by ILC2 and help the polarization of Th2 and Tfh cells from Th0. Th2 cells produce IL-4, IL-5 and IL-13 that drive class-switching of B cells for the production of sIgE. Tfh cells produce IL-4 that is also important for the production of sIgE. These IgE then binds to the FcεRI on MCs and basophils, leading to their sensitization. (**B**) Subsequent exposure to the same allergen, DCs recognize the allergen and drive the development of Th2 responses, which result in IgE-mediated FcεRI cross-linking on MCs and basophils. Consequently, preformed mediators such as histamine and proteases are released that contribute to the early-phase allergic responses. Newly synthesized mediators such as leukotrienes released by MCs and basophils as well as IL-5 produced by ILC2 and Th2 cells lead to the infiltration of eosinophils that are crucial for the late-phase allergic inflammation.

**Figure 2:**
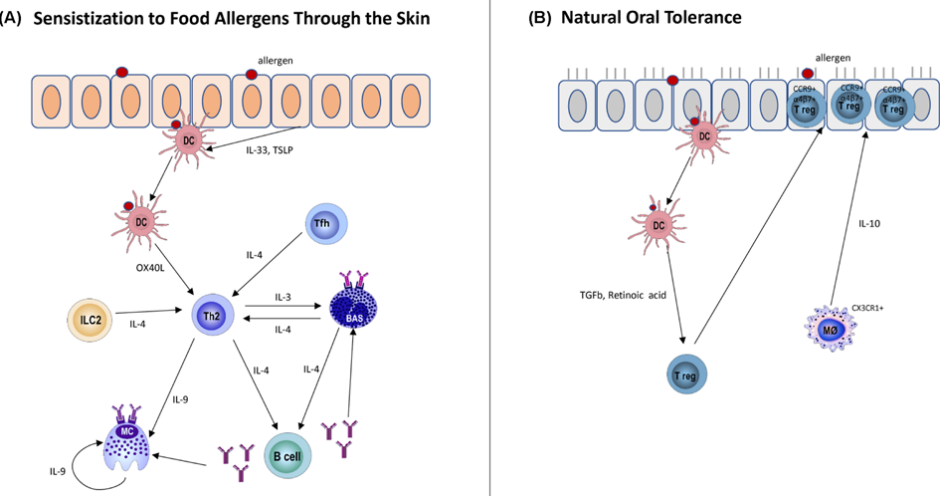
Immunological mechanisms of (A) skin sensitization and (B) natural tolerance to food allergens (**A**) Food allergen in the presence of trigger e.g. epithelial damage enhances the production of IL-33 and TSLP by epithelial cells and induces DC maturation. DC up-regulates OX40L and present antigens to T cells. Th2 response is induced and IL-4 produced by Th2 cells promotes the IgE class-switching on B cells. Tfh can also contribute to IgE-class-switching on B cells. Basophils can also enhance Th2 responses. IL-9 produced by Th2 cells is crucial for MC expansion. The IgE antibodies produced bind to the FcεRI on MCs and basophils, resulting in sensitization. (**B**) DCs capture antigen at the lamina propria and move to the lymph node where they induce Tregs through mechanisms that require TGF-β and retinoic acid. Gut-homing markers CCR9 and α4β7 are expressed by antigen-specific Tregs and migrate from the lymph nodes to the lamina propria and Tregs expand in response to IL-10 produced by CX3CR1^+^ macrophage.

Similar to macrophages, DCs have demonstrated functional plasticity under different conditions. In a study by Gueguen et al*.* [30], molecular markers for four different monocyte-derived DC subclasses (DC1, DC2, DC17 and DCreg) that stimulate the differentiation of Th1, Th2, Th17 or Tregs respectively, were identified through comparative transcriptomic and proteomic analyses. In the same study, 4 months after AIT for grass pollen, patients have up-regulated DCreg markers but down-regulated DC2 markers. Additionally, there was a significant increase in DCs numbers with DCreg phenotype (assessed by mRNA expression of stabilin-1 and complement component 1Q, C1Q) in the peripheral blood samples of responders four months after SLIT for grass pollen allergy comparing with that before the SLIT [29]. In support of this, children who were allergic to HDM and had undergone SLIT resulted in an immature phenotype for their peripheral DCs, with enhanced capacity to generate IL-10 with diminished IL-12 [180]. Additionally, both SLIT and OIT for cow’s milk allergy induce a pro-tolerogenic phenotype in DCs and co-culture of CD4^+^ T cells with plasmacytoid DCs from these AIT-treated patients resulted in reduced Th2 cytokine production after stimulation with cow's milk extract [181]. Further, the study by Palomares et al*.* [182] demonstrated up-regulation of programmed death-ligand 1 (PD-L1) in DCs after one year of treatment with SLIT for peach allergic patients and suggested that this could be the link to the increased number of Treg cells with higher PD-L1 expression and IL-10 production observed in these SLIT-treated patients. Therefore, ‘re-educating’ DCs from the pro-allergic phenotype to the regulatory profile represents a novel strategy for allergy treatment. Figure 1 summarizes the mechanism of IgE sensitization to a specific allergen and after re-exposure to the same allergen for AR (Figure 1) while Fig. 2 illustrates the impact of antigen exposure through the skin and oral route respectively (Figure 2).

## Next generations of allergen-specific immunotherapies

AIT, considered as precision medicine, should be provided to the right patients at the right dose and right time [7]. For instance, patients with IgE sensitivities to grass pollen profilin Phl p12 might lead to up-regulated sIgE production and false skin prick test to whole birch pollen due to the cross-reactivity with birch profilin Bet v2 while the patients with high IgE levels to Phl p 1 and Phl p5 might represent good candidates for grass pollen immunotherapy [6]. To better improve patient selection for AIT, more effort has been invested on the characterization of allergen extracts using transcriptomics and proteomics approaches and profiling of their IgE reactivity [183–185]. The development and use of purified recombinant allergen, fusion of several allergens or hypoallergenic allergen derivatives will allow standardization of the allergen as compared to currently used natural allergen extracts, thereby expanding our knowledge on the disease mechanism and improving diagnosis and AIT for allergies [186]. Further, there are clinical trials showing an improved safety profile of recombinant allergens than natural allergen extracts in AIT for AR [143,187] and the use of allergoid has also shown efficacy with better safety, probably through reduced basophil activation [188].

To improve the recombinant hypoallergens, recombinant B cell epitope-base vaccines are developed and these vaccines lack IgE reactivity and allergenic activity [189,190], had inhibited allergen-specific T-cell responses [189,191,192] but high immunogenicity through induction of IgG responses by the carrier protein [189,193] and thus has high efficacy but less adverse event. BM21, the vaccine for grass pollen contains non-IgE-reactive peptides derived from the IgE-binding sites of the grass pollen allergens and covalently linked to Pre S from hepatitis B virus, a viral protein carrier that provides carrier-specific T cell help was tested in clinical trials [189–191] and the phase 2b multi-centred, double-blinded, placebo-controlled study reported that BM21 can relieve allergy symptoms and is well tolerated without severe adverse effects [194,195].

Besides, several other novel strategies, with the aim to improve the efficacy and safety have been tested. These include but not limited to the use of adjuvants like monophosphoryl lipid A in combination with SCIT for AR to target immune deviation from Th2 to Th1 response [196], biologics like omalizumab (anti-IgE antibody) to inhibit MC and basophil functions or anti-IL-4 to dampen Th2 response, DNA vaccine to prevent Th2 response but promote Th1 response with better safety profile [152,189].

Since several studies have shown the correlation between composition and metabolic activity of the commensal microbiota and allergies or tolerance to food allergen, there is increasing interest in microbial therapeutics for FA. Peanut allergic patients treated with OIT and *Lactobaccillus rhamnosus* GG for 18 months have higher rate of desensitization compared with placebo controls but the efficacy of probiotic alone remains undetermined as there was no OIT-only or probiotic-only group included in the study [64,197].

In an attempt to reduce risk of adverse allergic reactions during AIT for FA, clinical trials have been investigating the combined administration of OIT with omalizumab and a phase I clinical trial confirmed that OIT with omalizumab is safer than OIT alone, with no severe adverse events being reported and rapid desensitization was also achieved [8,198]. Furthermore, Nadeau et al. [199] also showed that up-dosing of food allergens to reach a maintenance phase can be more rapid after addition of omalizumab to OIT and hence, combined administration of OIT and omalizumab has improved safety profile with retained efficacy [64].

However, majority of these studies were done *in vitro* or were small-scale pilot trials and hence larger-scale randomized controlled clinical trials are required to evaluate the efficacy and safety of these novel approaches [200]. On the other hand, thanks to exponential advances in the ‘omics’ technologies, longitudinal profiling of relevant cells in patients before, during and after AIT comparing with those who were prescribed with placebo and non-atopic individuals is now feasible and the dataset generated will allow us to further dissect the mechanisms of allergic diseases and AIT, and hence leading to better tailoring of therapies for allergic patients [201–205].

## Conclusion

**Figure 3:**
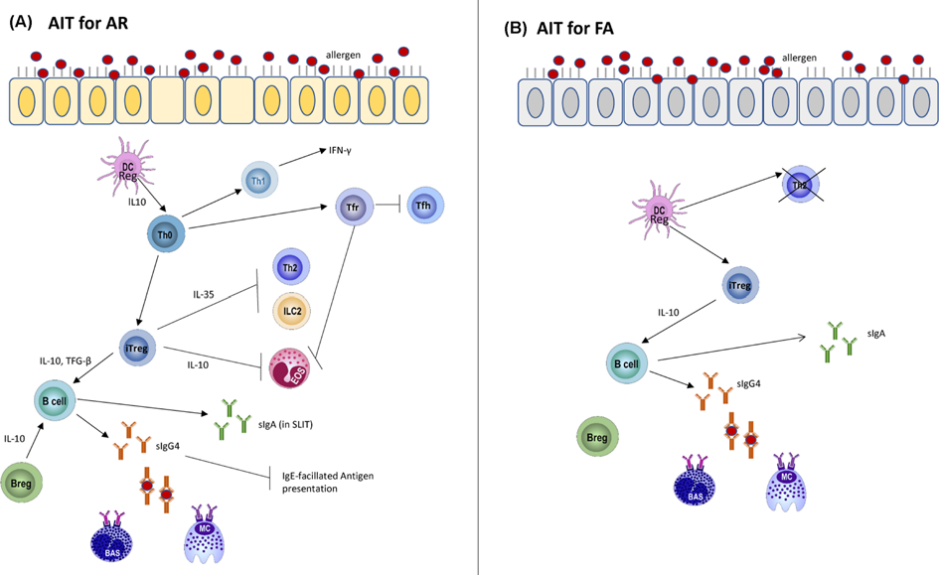
Mechanisms of immune tolerance to (A) inhalant and (B) food allergen induced after successful AIT (**A**) After high dose of allergen introduced during AIT, DCregs produce IL-10 and Th2 is shifted to Th1 responses. Bregs and Tregs are induced and they produce IL-10 while Tregs also produce TGF-β. Both IL-10 and TGF-β promotes class-switching on B cells to enhance production of blocking sIgG4 that compete with sIgE for allergen binding. Thus, the activations of MCs and basophils are inhibited. (sIgA are also produced in SLIT.) IL-10 produced by Tregs downregulates eosinophil functions while IL-35 produced by Tregs suppresses ILC2 and Th2 cells. Tfr differentiated inhibit eosinophils and Tfh cells. (**B**) Similarly, Tregs are induced after AIT for FA. IL-10 produced by Tregs lead to class-switching of B cells and thereby production of sIgG4 antibodies that prevent the cross-linking of the FcεRI on MCs and basophils. Besides sIgG4, sIgA are also produced. Further, Th2 cells undergo anergy or are deleted.

Taken together, AIT is the only potential disease-modifying therapy for allergic diseases including AR and FA, mainly through inducing a long-term tolerance (or remission in the case of FA) against the specific allergen. Successful immunotherapy for AR engages a complex alteration of the immune network and has been mainly associated with increased number of Tregs resulting in the elevated levels of IL-10 and TGF-β, reduced number of Th2 cells, shifting of Th2 to Th1 immune responses, probably due to the increased numbers of DCregs. Furthermore, increased level of sIgG4 blocking antibodies to inhibit MCs and basophils activation, modulated functions and reduced numbers of MCs, basophils and eosinophils as well as increased numbers of Bregs are reported after AIT for AR. In SLIT for AR, the level of serum sIgA is also enhanced. In the context of AIT for FA, the suppression of allergic symptoms is probably mediated by induction of Tregs or reduction of Th2 responses by anergy as a result of the induction of pro-tolerogenic phenotype in DCs, together with elevated levels of sIgG4 to block MCs and basophils activation and the production of sIgA and Bregs (see Figure 3). However, the precise molecular and immunological mechanisms for AIT remain to be elucidated. The ultimate goal is to improve the efficacy and safety of AIT, ideally with shorter treatment course and more convenient administration as well as the identification of reliable biomarkers that allow prediction of responders before or at the early phrase of the AIT. With the revolution of ‘omics’ technologies, novel strategies for AIT are highly expected to be achieved.

## Abbreviations

AIT: allergen-specific immunotherapy
AP-1: activator protein-1
AR: allergic rhinitis
Breg: regulatory B
CCL: C-C motif chemokine ligand
CCR: C-C motif chemokine receptor
CD4: cluster of differentiation 4
CLA: cutaneous lymphocyte antigen
CRTH2: chemoattractant receptor-homologous molecule expressed on Th2 lymphocytes
CX3CR: C-X3-C motif chemokine receptor
DC: dendritic cell
EPIT: epicutaneous immunotherapy
FA: food allergies
FcεRI: high-affinity receptor for immunoglobulin E
FOXP3: Forkhead box P3
GAP: goblet cell-associated antigen passage
HDM: house dust mite
IgE: immunoglobulin E
IL: interleukin
ILC2: Group 2 innate lymphoid cell
iTreg: inducible regulatory T
LT: leukotriene
MC: mast cell
NFAT: nuclear factor of activated T cells
NF-κB: nuclear factor-kappa B
nTreg: natural regulatory T
OIT: oral immunotherapy
PBMC: peripheral blood mononuclear cell
PD-L1: programmed death-ligand 1
PGD: prostaglandin
RALDH: retinaldehyde dehydrogenase
SCIT: subcutaneous immunotherapy
sIgA: allergen-specific IgA
sIgE: allergen-specific IgE
sIgG: allergen-specific IgG
SLIT: sublingual immunotherapy
Tfh: T follicular helper
Tfr: follicular regulatory T
TGF-β: transforming growth factor β
Th: T helper
TNFα: tumor necrosis factor α
Treg: regulatory T
TSLP: thymic stromal lymphopoietin protein
